# Fecal microbiota transplantation alters the susceptibility of obese rats to type 2 diabetes mellitus

**DOI:** 10.18632/aging.103756

**Published:** 2020-09-12

**Authors:** Lijing Zhang, Wen Zhou, Libin Zhan, Shenglin Hou, Chunyan Zhao, Tingting Bi, Xiaoguang Lu

**Affiliations:** 1 School of Traditional Chinese Medicine and School of Integrated Chinese and Western Medicine, Nanjing University of Chinese Medicine, Nanjing 210023, China; 2 Department of Emergency Medicine, Zhongshan Hospital, Dalian University, Dalian 116001, China

**Keywords:** intestinal microbiota and metabolites, obesity, type 2 diabetes mellitus, leptin receptor, susceptibility

## Abstract

Obesity is one of the susceptibility factors for type 2 diabetes (T2DM), both of which could accelerate the aging of the body and bring many hazards. A causal relationship is present between intestinal microbiota and body metabolism, but how the microbiota play a role in the progression of obesity to T2DM has not been elucidated. In this study, we transplanted healthy or obese-T2DM intestinal microbiota to ZDF and LZ rats, and used 16S rRNA and targeted metabonomics to evaluate the directional effect of the microbiota on the susceptibility of obese rats to T2DM. The glycolipid metabolism phenotype could be changed bidirectionally in obese rats instead of in lean ones. One possible mechanism is that the microbiota and metabolites alter the structure of the intestinal tract, and improve insulin and leptin resistance through JAK2 / IRS / Akt pathway. It is worth noting that 7 genera, such as *Lactobacillus*, *Clostridium* and *Roche,* can regulate 15 metabolites, such as 3-indolpropionic acid, acetic acid and docosahexaenoic acid, and have a significant improvement on glycolipid metabolism phenotype. Attention to intestinal homeostasis may be the key to controlling obesity and preventing T2DM.

## INTRODUCTION

The World Health Organization first released a diabetes report in 2016 that showed that type 2 diabetes mellitus (T2DM) has become a chronic worldwide disease [1]. T2DM is a chronic disease with a high incidence in the elderly and in which pathogenesis is intricate. Elderly people with T2DM are more likely to suffer from complications, and the treatment is more difficult [2]. Currently, the incidence of T2DM is greater than 25% in elderly patients over 65 years of age [3]. Therefore, more and more research is focused on the early diagnosis and treatment mechanism of T2DM [4, 5].

One of the main factors leading to the surge in T2DM is obesity [6, 7]. Insulin resistance (IR) is a key factor in obesity and T2DM. Increases in obesity-related immune activation [8] and circulating leptin levels [9] induce systemic IR, which greatly increases susceptibility to T2DM. Therefore, controlling obesity, improving IR and leptin resistance (LR), and delaying or reversing the occurrence of T2DM are major goals that need to be addressed urgently.

Growing evidence suggests a link between the intestinal microbiome and the metabolic health of the human body [10–13]. In 2016, a breakthrough study by a European and Chinese team found that specific intestinal microbiota imbalances lead to IR, leading to an increased risk of health problems such as T2DM [14]. Some evidence suggests that the IR phenotype can be transferred by transplanting the fecal microbiome [15–17]. To demonstrate this benefit, distinction between the characteristics of the microbiome that cause the disease and the characteristics of the disease or its therapeutic consequences is necessary [18]. Changes in *Ruminococcus sp.*, *Escherichia coli*, *Bacteroides*, and *Akkermansia muciniphila* have been observed in diabetic and obese patients [19–22]. These different results suggest that a better understanding of the role of specific taxa in regulating host metabolic function is needed. Changes in the abundance of these microbiota, such as *A. muciniphila*, *Proteus mirabilis*, or *Bacteroides uniformis*, are associated with the glucose metabolism pathway. The proposed hypothesis of key microbial-phenotypic associations necessitates future research on microbiota [23]. Studies from Germ Free (GF) mice have further demonstrated that the intestinal microbiota is the cause of glucose intolerance caused by high-fat diets [24–26], and that obesity can be metastasized via fecal microbiota transplantation (FMT) [27]. These studies suggest that the progression from obesity to IR to T2DM is accompanied by changes in the species of the intestinal microbiota, and the imbalance occurs before the disease occurs.

The possible mechanisms of T2DM induced by the intestinal microbiota include disorders of lipid metabolism, endotoxemia, bile acid metabolism, insulin resistance etc. In order to better understand the role of intestinal microbiota in the obesity-T2DM process and the possible mechanism, we used Zucker Diabetic Fatty (ZDF) rats with mutations in the leptin receptor gene as a research model, which can gradually produce spontaneous obesity and hyperglycemia with age. In this process, FA mutation causes the leptin receptor to shorten, and a large amount of free leptin cannot bind to the corresponding receptor to exert its role. This mutation is phenotypically manifested as obesity with high leptin levels in the blood. The phenomenon of high serum leptin levels coexisting with obesity and abnormal glycolipid metabolism is leptin resistance, and leptin resistance is also observed in obese people. Along with the increase of age, the disorder of leptin signal and insulin signal transduction is aggravated, which can develop into T2DM [28]. Continuous FMT of obese-T2DM models' microbiota could change the phenotype of glycolipid metabolism, microbiota composition, metabolite structure and colon pathological characteristics of recipient rats during the development from obesity to T2DM. There were also differences in leptin and insulin signaling pathways at the molecular level. At the same time, in order to comprehensively analyze the relationship between host phenotype, intestinal microbiota and its metabolites, we generated correlation matrix by calculating Spearman correlation coefficient to determine the significant effect of the latter two on the former. This study explored the effects of changes in gut microbiota on normal or leptin receptor gene deficiency rats, and multi-angle analysis of the directivity of intestinal microbiota during the progression of obesity to T2DM.

## RESULTS

### FMT altered the glycolipid metabolism phenotype in ZDF rats

**Figure 1:**
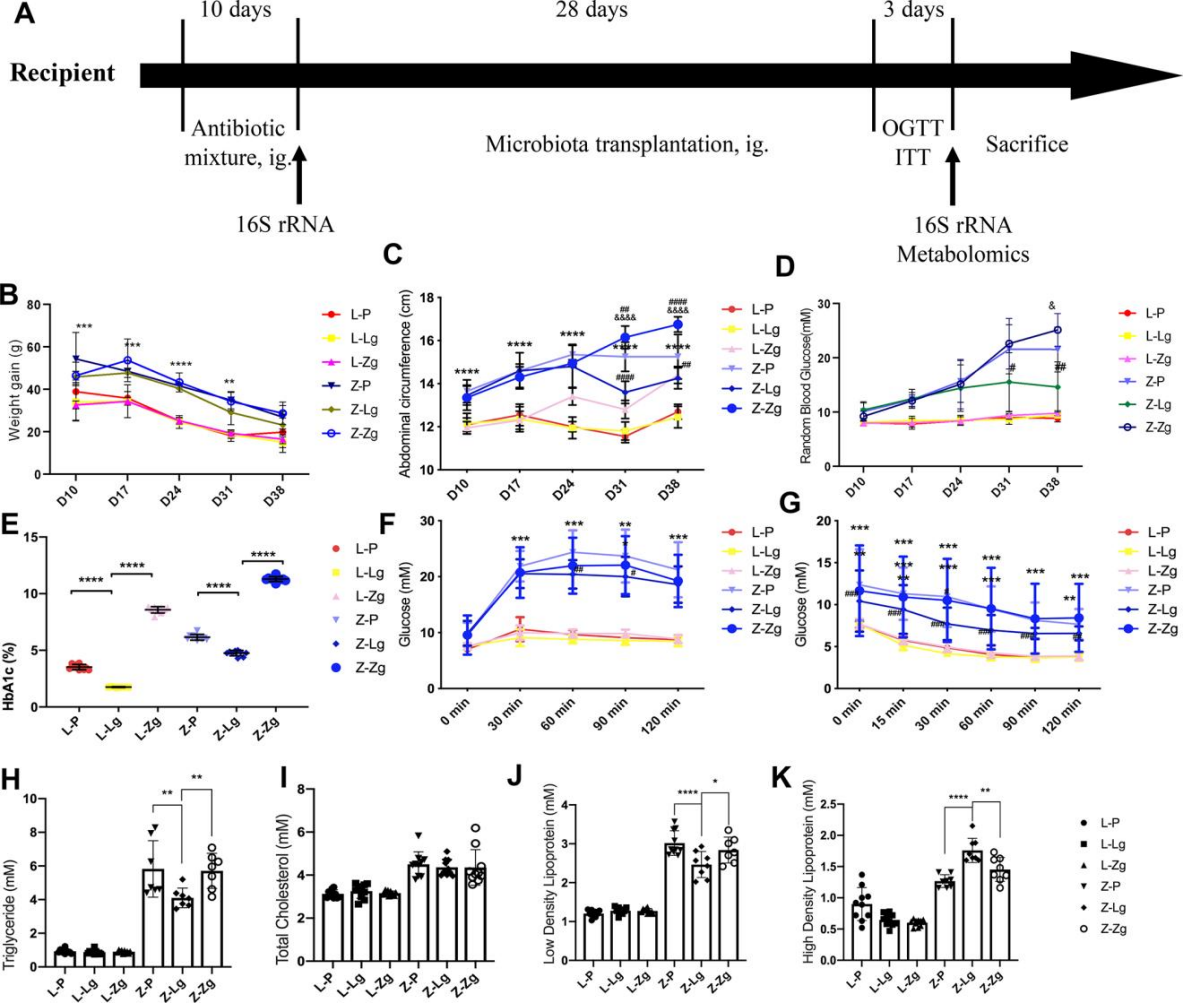
**Changes in glycolipid metabolism phenotypes in recipient rats before and after transplantation.** (**A**) Detailed information. LZ rats were fed a normal diet, and ZDF rats were fed an induced diet #5008. After adaptive feeding, the four groups were given an antibiotic mixture for 10 days, and then the corresponding supernatant from the LZ group and ZDF group was given to LZ and ZDF recipient rats, whereas the control group was given PBS. The course of T2DM was judged by OGTT, ITT, RBG, and FSI. After antibiotic administration and FMT, feces were collected for 16S rRNA sequencing and metabolomic analysis of intestinal contents at the end of the experiment; (**B**) Weight gain at different stages (g; Time: F_3, 115_ = 90.60, *P* < 0.0001; Group: F_4, 45_ = 110.2, *P* < 0.0001; Interaction: F_20, 190_ = 1.844, *P* < 0.05; n = 7-10); (**C**) Abdominal circumference at different stages (cm; Time: F_5, 270_ = 318.0, *P* < 0.0001; Group: F_4, 270_ = 67.39, *P* < 0.0001; Interaction: F_20, 270_ = 16.09, *P* < 0.0001; n = 10); (**D**) Random blood glucose at different stages (mM; Time: F_2, 90_ = 131.3, *P* < 0.0001; Group: F_4, 45_ = 55.78, *P* < 0.0001; Interaction: F_20, 214_ = 12.67, *P* < 0.0001; n = 8-10); (**E**) Glycosylated hemoglobin after FMT (%; F_5, 54_=2396, P < 0.0001; n = 10); (**F**) Comparison of OGTT (mM; Time: F_4, 216_ = 190.3, *P* < 0.0001; Group: F_5, 54_ = 55.93, *P* < 0.0001; Interaction: F_20, 216_ = 20.59, *P* < 0.0001; n = 10); (**G**) Comparison of ITT (mM; Time: F_5, 270_ = 134.4, *P* < 0.0001; Group: F_5, 54_ = 11.58, *P* < 0.0001; Interaction: F_25, 270_ = 1.942, *P* = 0.0056; n = 10); (**H**) The levels of TG (mM; F_5, 45_ = 84.27, *P* < 0.0001); (**I**) TC (mM; F_5, 54_ = 20.55, *P* < 0.0001); (**J**) LDL-C (mM; F_5, 49_ = 131.0, *P* < 0.0001), and (**K**) HDL-C (mM; F_5, 48_ = 68.74, *P* < 0.0001) after FMT (mM, n = 7-10). *P < 0.05, **P < 0.01, and ***P < 0.001 vs. L-P, ^#^*P* < 0.05, ^##^*P* < 0.01, and ^###^*P* < 0.001 vs. Z-P, ^&^*P* < 0.05 vs. Z-Lg in (**B**–**D**, **F**, **G**). *P < 0.05, **P < 0.01, ***P < 0.001, and ****P < 0.0001 indicated inter-group changes in (**E**) and (**H**–**K**). Statistical analysis was performed with two-way ANOVA in (**B**–**D**), one-way ANOVA in (**E**) and (**H**–**K**) and repeated ANOVA in (**F**, **G**). The data were expressed as the mean ± SD.

In the donor group, all metabolic evaluation indicators showed that the obese T2DM model was successfully induced in the ZDF group. These metabolic indicators were significantly different from the LZ group (Supplementary Figure 1A–1I). The experimental procedure was implemented as shown in Figure 1A. The group of LZ rats receiving LZ intestinal microorganisms was named L-Lg, receiving ZDF intestinal microorganisms was named L-Zg, and receiving PBS was named L-P. The ZDF recepient group was named the same way. Each transplantation group was given by gavage with a mixed antibiotic solution from D1-D10, and then transplanted the microbiota for 4 weeks. Record the corresponding data every week. From the third week of FMT, the metabolic characteristics of ZDF rats showed significant changes in response to transplantation. Figure 1B recorded the changes in the weight gain of the rats at different stages. The results showed that with the natural growth of the rats and the prolongation of the transplantation time, each group showed a slowing trend of the growth rate. For the LZ recipient rats, the weight gain had no changes in response to microbial transplantation, however, in ZDF recipient rats, the weight gains of rats transplanted with normal microbiota decreased significantly, while the weight gain of rats transplanted with obesity-T2DM microbiota had an increasing trend, and the abdominal circumference of the Z-Zg group increased significantly (*P* < 0.0001). The random blood glucose in the Z-P and Z-Zg groups was significantly higher than that in the Z-Lg group (*P* < 0.05), and the difference was more significant at 38 days (*P* < 0.001). Glycated hemoglobin was a good indicator of intra-group differences (Figure 1B–1E). These responses were not shown in LZ rats. The levels of glycated hemoglobin in the Z-P and Z-Lg groups showed a slightly lower trend than the L-Zg group, which seemed to be different from the level of random blood glucose. This may be due to the fact that random blood glucose responds to the immediate level of blood glucose and glycated hemoglobin reflects the average blood glucose level for a long time before the blood collection point, which is more stable and is not disturbed by the activities. The results of glycated hemoglobin levels in the L-Zg, Z-P and Z-Lg groups reflected that the blood glucose did not reach the state of hyperglycemia during 5-8 weeks of age, which was consistent with the natural growth of ZDF rats and the performance of random blood glucose. The higher level of glycated hemoglobin in L-Zg group could also reflect that although the short-term T2DM microbiota transplantation did not induce healthy LZ rats to form stable obesity and type 2 diabetes, but the rats also showed a corresponding trend. However, combined with body weight, OGTT, ITT and insulin-related levels, LZ rats still maintained a relatively stable health status. Changes in blood lipids indicated that Z-Lg group were superior to the Z-P group (Figure 1H–1K). OGTT and ITT showed that glucose tolerance and insulin tolerance were better in the Z-Lg group than in the Z-P and Z-Zg groups, suggesting that transplantation of the LZ intestinal microbiota improved the insulin resistance of ZDF rats (Figure 1F, 1G).

### Effects of FMT on intestinal communities in rats

**Figure 2:**
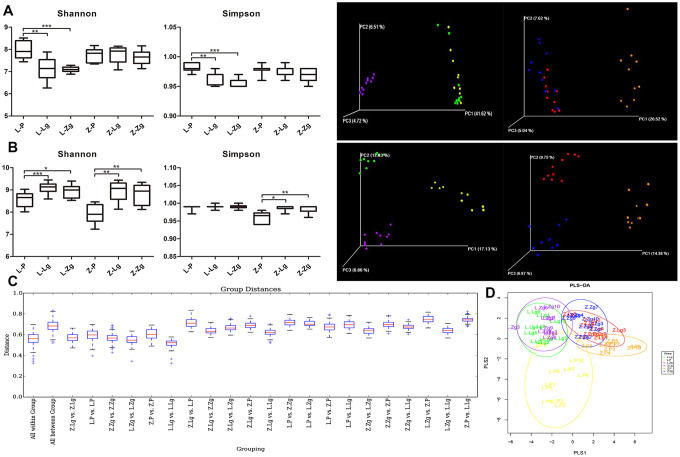
**Establishment of the pseudoaseptic rat model and evaluation of intestinal microbiota structure after FMT.** (**A**) Shannon index and Simpson index among six groups after intragastric administration of antibiotics and a three-dimensional sequence plot of unweighted UniFrac PCoA analysis corresponding to LZ and ZDF rats after antibiotics (Shannon: F_5, 52_ = 10.03, *P* < 0.0001; Simpson: F_5, 50_ = 12.94, *P* < 0.0001; n = 10); (**B**) Shannon index and Simpson index among six groups after FMT (Shannon: F_5, 53_ = 13.48, *P* < 0.0001; Simpson: F_5, 53_ = 14.69, *P* < 0.0001; n = 10) and a three-dimensional sequence plot of unweighted UniFrac PCoA analysis corresponding to LZ and ZDF rats after FMT (n = 10). The percentage in parentheses of coordinate axes represented the proportion of differences in the original data that the corresponding principal coordinates could explain. Statistical analysis was performed with one-way ANOVA in (**A**, **B**). *P < 0.05, **P < 0.01, and ***P < 0.001. The data were expressed as the mean ± SD; (**C**) Unweighted UniFrac distance box plots. Horizontal coordinates corresponded to statistical comparisons between groups and within groups, and longitudinal coordinates indicated the corresponding distance values. Borders of boxes represented the interquartile range (IQR), horizontal lines represented the median value, and upper and lower whiskers represented 1.5 outside the upper and lower quartiles. In the IQR range, the symbol “+” denoted potential outliers that exceed the range. Statistical analysis was performed with Student’s t-test and Monte Carlo permutation test; (**D**) PLS-DA discriminant analysis graph. Each point represented a sample. The same color points belonged to the same grouping, and the same grouping points were marked with ellipses (n = 10). Yellow: L-P; Green: L-Lg; Purple: L-Zg; Orange: Z-P; Red: Z-Lg; Blue: Z-Zg.

The response to FMT in the obese context is closely related to the baseline composition of the microbiota [15]. The metabolic response to FMT can be predicted by the baseline composition of the microbiota of the recipient [16]. There was no significant difference in diversity between the two groups of antibiotics administered to LZ and ZDF recipient rats (Figure 2A). Principal coordinates analysis (PCoA) showed that there was no significant difference in the spatial distribution of community samples between groups after antibiotic gavage, and there were significant differences between groups without antibiotic gavage (Figure 2A). This indicated that the pseudo-sterile rat model was successfully established, and the baseline of the intestinal microbiota of the recipient rats was the same.

The intestinal community identification of donor rats showed that the α-diversity index and β-diversity index between the two groups were significantly different (Supplementary Figure 2A–2C). After FMT, Shannon index and Simpson index showed that Z-P was lower than Z-Lg and Z-Zg (*P* < 0.05) in the recipient group. Unweighted UniFrac PCoA analysis and UniFrac distance value difference box plots showed that there were significant differences between the groups, indicating that the intestinal microbial system has changed after FMT (Figure 2B, 2C). PLS-DA (Partial Least Squares Discriminant Analysis) showed that the L-Lg and L-Zg communities had a higher degree of aggregation, and the Z-Lg, Z-Zg, and Z-P groups had better separation (Figure 2D).

**Figure 3:**
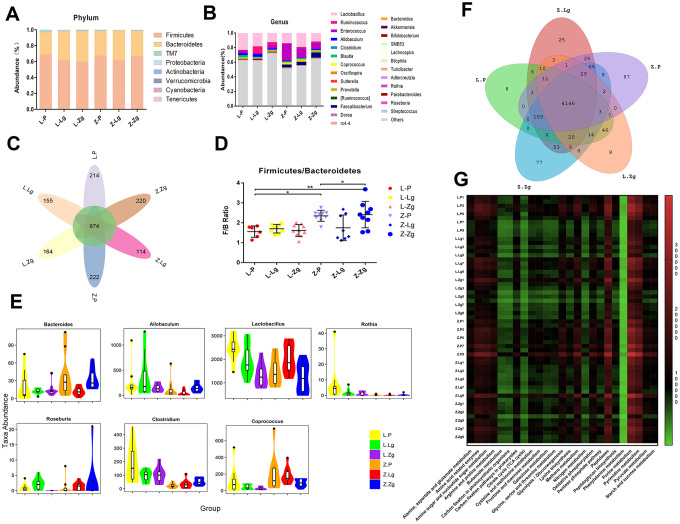
**Specific phyla and genera in each group after FMT.** (**A**) Relative abundance of bacteria at the phylum level (n = 10); (**B**) Relative abundance of bacteria at genus level (n = 10); (**C**) The petal diagram revealed common and unique genera associated with different groups. Different colors represented different modules; (**D**) *Firmicutes* / *Bacteroidetes* ratio (F_5, 45_ = 6.511, *P* = 0.0001; n = 8-9). Statistical analysis was performed with two-way ANOVA. *P < 0.05, **P < 0.01. The data were expressed as the mean ± SD; (**E**) Violin maps of abundance distribution of seven OTUs with the most significant difference among sample groups. The abscissa represented the group, and the ordinate represented the number of sequences of each taxon in each sample (group) (n = 10). Using Mothur software, the statistical algorithm of Metastats was invoked to test the difference in sequence quantity (absolute abundance) between the samples (groups) of each taxon at the phylum and genus levels; (**F**) The venn diagram of common functional groups predicted by PICRUSt. Each ellipse represented a sample (group). The overlapping regions between ellipses indicated common functional groups among the samples (groups). The number in each block indicated the number of common or unique functional groups of the samples (groups) included in the block; (**G**) KEGG third-level pathway heat map predicted by PICRUSt. The abscissa was the third level functional group of KEGG, and the ordinate was the sample number. The color markers were the number of macrogenomes constructed from biom files. The intensity of the colors represented the degree of association (red, higher number of corresponding samples; green, lower number of corresponding samples).

Different proportions of phyla were seen after FMT, of which more than 90% of the readings belonged to four phyla, *Firmicutes*, *Bacteroidetes*, *TM7*, and *Proteobacteria*. At the genus level, 26 categorical genera such as *Lactobacillus*, *Ruminococcus*, *Enterococcus*, and *Allobaculum* accounted for the major abundance (Figure 3A, 3B). In order to more clearly judge the diversity of microbiota composition between groups, we further used petal maps to show common and unique genera related to FMT. Different colors represented different modules. The petal map (node) in the center was shared by all groups, with a total of about 974 OTUs, which allowed us to see the different OTUs of each receptor group more clearly. The Z-P and Z-Zg groups showed more unique OTUs than that of LZ recepient rats, while LZ rats transplanted with ZDF microbiota showed a decrease in OTU, and ZDF rats transplanted with LZ microbiota showed an increase in OTU. This result further proved that the ZDF groups were more diverse than the LZ groups (Figure 3C). At the same time, *Firmicutes* / *Bacteroides* (*F* / *B*) ratio in the Z-Zg group increased significantly (Figure 3D). Consistent with the glycolipid metabolism phenotype, these changes did not respond to LZ recipient rats. The 16S rRNA gene sequence was conducted to determine identity. Metastats pairwise comparison test was performed according to the composition and sequence distribution of each sample at each taxonomic level, and the difference in sequence quantity between the samples (groups) of each taxon at the phylum and genera levels. Along with the invasion of the ZDF rats’ microbiota and the intensification of T2DM symptoms, *Bacteroides* showed high expression, while *Lactobacillus*, *Roseburia*, *Coprococcus*, *Rothia*, and *Allobacum* decreased, showing the opposite trend in the group of transplanted with LZ microbiota (Figure 3E). This was different from the differences identified in the donor group (Supplementary Figure 2A–2D and Supplementary Table 1). The microbes were interdependent and mutually antagonistic, maintaining the intestinal environment in a stable ecology and thus maintaining the health and stability of the body. However, the coordination mechanism between them is not completely understood. PICRUSt predicts the 16S rRNA gene sequence in the KEGG PATHWAY (http://www.genome.jp/kegg/pathway.html) database to obtain annotation information corresponding to each functional spectrum database for each sample. According to the abundance distribution of each functional group in each sample, R-software was used to calculate the number of common functional groups in each group, and the proportion of the functional groups shared and unique by each group was visually represented by a Venn diagram. The KEGG third-level pathway statistics showed that the microbiota results predicted that 25 pathways had significant changes, including nine in the carbohydrate pathway, seven in the amino acid metabolic pathway, five in the energy metabolism pathway, two in synthesis and metabolism, two in nucleotide metabolism and one in the enzyme family (Figure 3F, 3G).

According to obesity and T2DM disease progression and microbiota changes in LZ and ZDF rats, the control group LZ rats did not show a significant response to the transplanted LZ or ZDF rats microbiota, which was different to that in ZDF rats. It is speculated that the susceptibility of ZDF rats to obesity and T2DM is increased. On this basis, the change of the microbiota structure can inhibit or promote the symptoms with the FMT, which adds evidence for the directional role of intestinal microbiota in the progression of T2DM.

### FMT changed the pathological structure and insulin/leptin signaling pathway in ZDF rats

**Figure 4:**
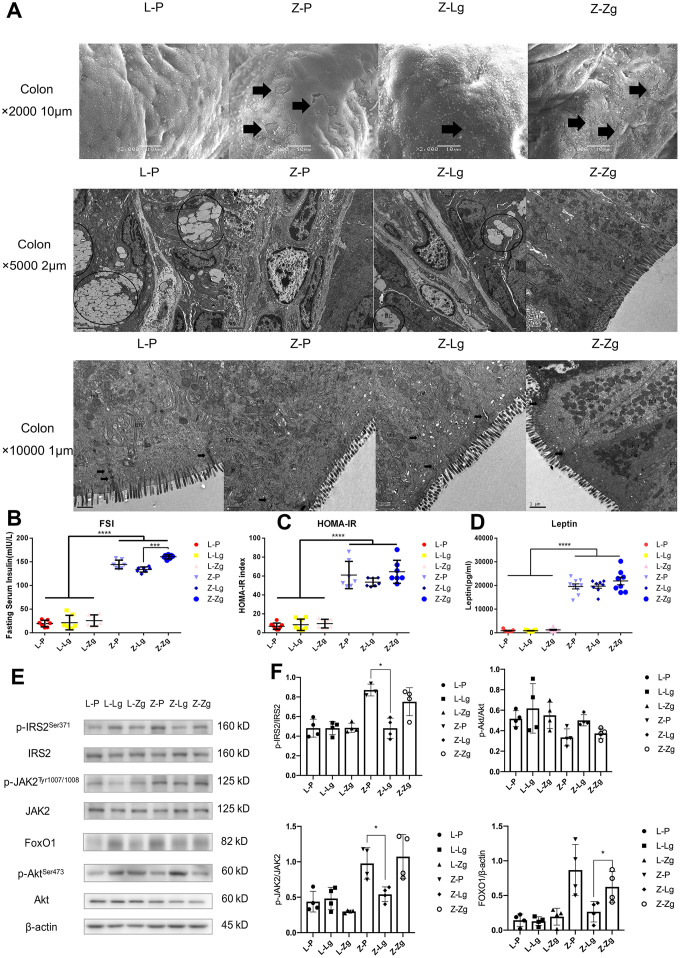
**The effects of FMT on the intestinal pathological structure, IR, and LR in rats.** (**A**) The colon surface of rats was magnified 2000 times (upper), 5000 times (middle), and 10,000 times (lower). IV: mucosal layer, microvilli on cell surface. mit: mitochondria. N: nucleus. BC: goblet cells. White vesicle structures were secretory vesicles. ER: endoplasmic reticulum; (**B**) Fasting serum insulin (mIU / L; F_5, 36_ = 351.5, *P* < 0.0001); (**C**) HOMA-IR Index (F_5, 39_ = 85.58, *P* < 0.0001); (**D**) Leptin in 6 groups (pg / mL; F_5, 43_ = 141.7, *P* < 0.0001; n = 7-9); (**E**) Western blotting analysis of IR and LR signaling pathway molecules in liver tissues was performed after FMT; (**F**) Quantification of western blotting analysis in (**D**) (p-IRS2 / IRS2: F_3, 15_ = 2.175, *P* = 0.0132; p-JAK2 / JAK2: F_3, 15_ = 0.5387, *P* = 0.6630; p-Akt / Akt: F_3, 15_ = 7.221, *P* = 0.0032; FoxO1 / β-actin: F_3, 15_ = 6.224, *P* = 0.0059; n = 3-4). Statistical analysis was performed with two-way ANOVA. *P < 0.05, **P < 0.01. The data were expressed as the mean ± SD.

According to the experimental results of glycolipid metabolism phenotype and intestinal microbiota, when LZ rats were used as recipients, no matter whether the normal or T2DM microbiota was transplanted, the symptoms and microbial structure did not show significant changes compared with the control group. It could be speculated that healthy hosts were more powerful in regulating the balance in the body, even if their gut community composition were changed to a certain extent, they could still restore themselves and return to health. Therefore, when we delved into the directional role of microbial transplantation in disease progression, we chose the ZDF recipient group that was more significant in response to FMT. In order to investigate the relationship between the intestinal tissue structure and the changes in intestinal microbiota, we examined the colonic pathological characteristics of four groups of rats with scanning and transmission electron microscopy. In the L-P group, microvilli were orderly and undamaged, and the number of goblet cells was high, the tight junction between cells was complete and compact, and mitochondria were high in number, large in volume, and complete in structure. In the Z-P group, typical microvilli were damaged and shed, and the number of goblet cells was reduced, mitochondria were swollen and cristae were arranged disorderly. In the Z-Lg group, the damage was less, but still existed while the most serious mucosal damage was found in Z-Zg group (Figure 4A). At the same time, fasting serum insulin (FSI) level showed that Z-Lg was lower than Z-Zg (*P* < 0.001) (Figure 4B–4D). The integrity of intestinal barrier is very important to health [29, 30] and one of the characteristics of obesity and T2DM is the damage of intestinal structure and barrier function [31, 32]. With the age increasing, the random blood glucose of ZDF rats continued to increase, but FSI and leptin were also high, indicating the presence of IR and LR. The liver is the key gatekeeper for draining intestinal blood from the portal vein. Even in a healthy state, the liver is often challenged by metabolic stress from gut microbiota and their metabolites. A complete intestinal epithelial barrier protects the liver from enormous bacterial exposure [33]. After the intestinal barrier is damaged, bacterial translocation and endotoxins enter the portal vein system, causing immune damage and inflammation, damage to distal organs, and impairment of the function of the body in multiple ways. The liver is the main peripheral target tissue for leptin and insulin, which regulate glucose metabolism. Transplanting the microbiota of LZ rats decreased p-JAK2 in the liver, and the expression of FoxO1 was inhibited by IRS / Akt pathway, while transplanting the microbiota of ZDF rats showed the opposite (Figure 4E, 4F). In other words, transplanting the microbiota of thin control rats can reduce the glucose metabolism dysfunction due to gene defects by regulating insulin resistance and leptin resistance, while transplanting the microbiota of obese T2DM rats will aggravate IR and LR.

### FMT changed intestinal metabolic characteristics of ZDF rats

**Figure 5:**
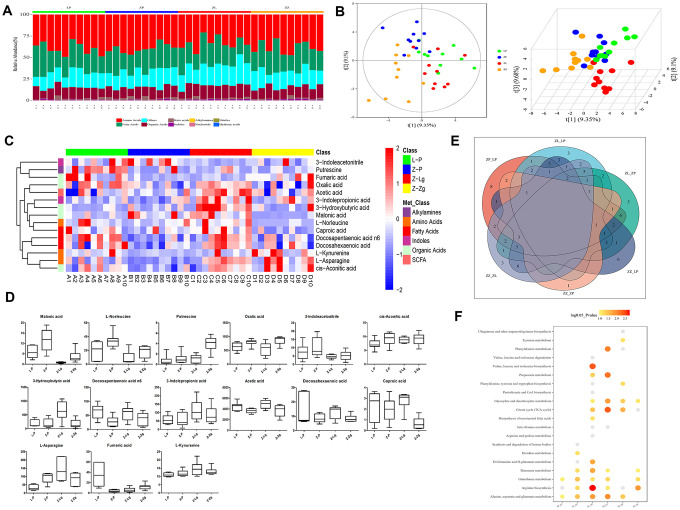
**Metabolite composition of intestinal microbiota in ZDF rats after FMT.** (**A**) The composition of metabolite types in each sample; (**B**) Score plot of 2D and 3D PLS-DA (n = 10). The green dots indicated L-P, the blue dots indicated Z-P, the red dots indicated Z-Lg, and the orange dots indicated Z-Zg; (**C**) Z-score heat map of differential metabolites. In the figure, the horizontal direction represented samples, and the longitudinal direction represents metabolites. The intensity of the colors represented the degree of association (red, higher content in the corresponding samples; blue, content in the corresponding samples. The relative numerical values represented by the colors were shown in the ribbon on the right.); (**D**) According to the results of single-dimensional statistics, the *P*-value was statistically significant for 15 groups of different metabolites as shown in box plots (n = 7-10); (**E**) Venn diagram of different metabolites. The number of shared and unique different metabolites screened by each group was shown; (**F**) Bubble map of the *P*-value of the metabolic pathway involved in the different metabolites. When the bubble was larger or the color was darker, the corresponding *P* value was smaller. Gray bubble, 0.05 < *P* < 0.1, Colored bubble, *P* < 0.05.

FMT altered the structure and characteristics of rat intestinal microbiota metabolites (Figure 5A, 5B). In order to determine the differences and similarities between the metabolite profiles in different samples, all the differential metabolites were clustered naturally. The results showed that the Z-P and Z-Zg groups had specific clustering effects among some metabolites, such as 3-hydroxybutyric acid, docosahexaenoic acid, 3-indolepropionic acid, L-Norieucine, Malonic acid, etc., which were significantly different from the L-P or Z-Lg group (Figure 5C). Orthogonal Partial Least Squares Discriminant Analysis (OPLS-DA) verifies that the model was credible (Supplementary Figure 3). In order to visualize the differences between the metabolites in the group, a one-dimensional statistical analysis was performed to obtain the top-ranked (*P* < 0.05, Table 1) representative differential metabolites as boxplots (Figure 5D). Among them, after transplanting the microbiota of LZ rats, the metabolites were transformed into the metabolic structure of the L-P control group, and the metabolic direction was completely changed after the microbiota of ZDF rats was transplanted. The number of shared and unique metabolites for each set of screens was shown as a Venn plot (Figure 5E). Based on different metabolite analysis, Metabolite Pathway Enrichment Analysis (MPEA) can classify the metabolic pathways involved using *P* values and mathematical algorithms. The 6 pathways of Citrate cycle (TCA cycle), Synthesis and degradation of ketone bodies, Butanoate metabolism, Phhenylanine metabolism, Beta-Alanine metabolism, Alanine, aspartate and glutamate metabolism were consistent with the results of the aforementioned microbiota prediction pathway.

**Table 1:** Different metabolites based on one-dimensional statistical analysis.

**No.**	**Name**	**Class**	**HMDB**	***P-*value**
1	Malonic acid	Organic Acids	HMDB00691	6.20E-05
2	L-Asparagine	Amino Acids	HMDB00168	1.80E-03
3	L-Norleucine	Amino Acids	HMDB01645	2.90E-03
4	Fumaric acid	Organic Acids	HMDB00134	5.40E-03
5	Putrescine	Alkylamines	HMDB01414	6.00E-03
6	3-Hydroxybutyric acid	Organic Acids	HMDB00357	7.40E-03
7	L-Kynurenine	Amino Acids	HMDB00684	7.80E-03
8	Docosapentaenoic acid n6	Fatty Acids	HMDB01976	1.40E-02
9	3-Indolepropionic acid	Indoles	HMDB02302	2.00E-02
10	Acetic acid	Fatty Acids	NA	2.40E-02
11	Oxalic acid	Organic Acids	HMDB02329	2.80E-02
12	Docosahexaenoic acid	Fatty Acids	HMDB02183	3.00E-02
13	Caproic acid	Fatty Acids	HMDB00535	3.40E-02
14	3-Indoleacetonitrile	Nitriles	HMDB06524	4.70E-02
15	cis-Aconitic acid	Organic Acids	HMDB00072	4.70E-02

### Potential relationship among host phenotypes, intestinal microbiota, and metabolites

**Figure 6:**
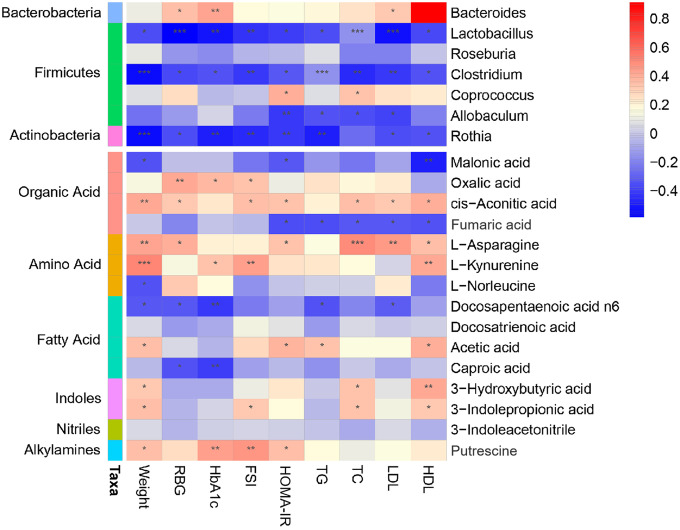
**Association map of the three-tiered analyses integrating the gut microbiome, phenotypes, and metabolome.** The left side of the panel showed associations between gut microbiota and phenotypes. The right side of the panel showed associations between metabolites and phenotypes. The intensity of the colors represented the degree of association (red, positive correlation; blue, negative correlation). *P < 0.05, **P < 0.01, ***P < 0.001.

To comprehensively analyze the relationship among the host phenotype, the intestinal microbiota, and the intestinal microbial metabolites, a correlation matrix was generated by calculating the Spearman correlation coefficient (Figure 6). In the obesity-T2DM process, three genera, *Lactobacillus*, *Clostridium*, and *Rothia*, showed a negative correlation with all phenotypes and might be an effective intervention for delaying the progression. *Lactobacillus* can regulate the balance of serum lipids, glucose, etc. through bile acids, benzene derivatives, organic acids, and others to promote lipid absorption, maintain intestinal barrier function transport, and conduct endocrine function signals; *Clostridium* can affect intestinal permeability through bile acids, lipids, etc. and activate the intestinal-brain-hepatic nerve axis to regulate glucose balance; *Rothia* can provide energy to the colonic epithelium by producing SCFAs and participate in the progression of obesity, insulin interference, and T2DM. *Allobaculum* is more closely related to obesity indicators. We further demonstrated that 3-hydroxybutyric acid, docosahexaenoic acid, n6, 3-indolepropionic acid, acetic acid, docosahexaenoic acid, and hexanoic acid were significantly increased; these may be key metabolites that can delay the progression of obesity to T2DM. Fumaric acid was negatively correlated with HOMA-IR and other blood lipid indicators, and may be an effective substance for obesity control. Fatty acids such as docosapentaenoic acid N6 and caproic acid were negatively correlated with blood glucose and glycosylated hemoglobin, and may be closely related to a delay in progression to T2DM. The mechanisms of fatty acids [34–36] and amino acids [14, 37–39] and their derivatives [40–42] are being explored. The intestinal-insulin axis formed by the host and microbiota during symbiotic evolution regulates the insulin level [43], which confirmed that there is a close relationship between microorganism and host in the course of obesity-T2DM.

## DISCUSSION

Aging is one of the causes of abnormal glucose metabolism [44]. It has been shown that age-related glucose intolerance, and even hyperglycemia, can disrupt the stability of glucose metabolism, leading to the onset of T2DM [45]. Given that dysfunctional leptin signaling is highly correlated with metabolic diseases such as obesity and T2DM [46], we used ZDF rats as the T2DM model. With the increase of age, the obesity and blood glucose levels of ZDF rats continue to increase, accompanied by severe insulin resistance, and even with the increase of blood glucose, a series of complications related to T2DM gradually appear, which are related to aging. The clinical manifestations observed in phenotypic individuals are consistent.

In humans, FMT can be seen as a tool to separate associations from the causality of multiple diseases [47]. At present, it has been recommended by many clinical medical guidelines and consensus for the treatment of refractory Clostridium difficile infection (CDI). In addition, FMT is used in the treatment of inflammatory bowel diseases (IBD), irritable bowel syndrome (IBS), functional constipation (FC) and autism. Various clinical studies have also shown certain efficacy [48–49]. For example, the clinical remission rate of FMT for active ulcerative colitis is 24% to 32%, and its symptom relief is related to the specific intestinal microbiota and abundance of metabolites [50–55]. FMT treatment of Crohn’s disease has shown an average clinical remission rate of 47% to 52% [55]. These therapeutic potentials are attributed to restoring intestinal microbial balance by replacing pathogens with more beneficial bacteria [56]. In addition to intestinal diseases, researchers are currently focusing on metabolic diseases, nervous system diseases and cardiovascular diseases. T2DM is closely related to the imbalance of intestinal microbiota. The change of intestinal microbiota is one of the most important environmental factors that promote the development of T2DM [57]. The composition of the intestinal microbiota can be beneficially modified by microbial-based therapies to maintain glucose homeostasis. A study showed that a double-blind randomized controlled trial in men with insulin resistance was conducted who received autologous or allogeneic fecal transplantation from thin donors, and obesity and insulin resistance were significantly improved [15–16]. Other clinical trials are needed to verify the effect of FMT on patients with insulin resistance and T2DM. At present, researchers at Nanjing Medical University in China have evaluated a 2-year clinical trial, one of which is the result of a phase 3 clinical trial of FMT performed on T2DM by FMT under nasal gastroscope. Other clinical trial studies on the effect of FMT on T2DM is being studied [58].

Our study showed that the IR phenotype, intestinal microbiota structure, and metabolic profiles of leptin receptor-deficient mice could be transferred with FMT, and that this transferable trait was not realized in control non-mutant mice. The microbiota structure and metabolic spectrum corresponding to worse symptoms changed negatively. The beneficial bacteria producing Short Chain Fatty Acids (SCFAs) such as *Lactobacillus*, *Rothia*, *Roseburia*, and *Coprococcus* decreased, and the metabolites of 3-hydroxybutyric acid, n-6,3-indolepropionic acid, acetic acid, docosahexaenoic acid, and hexanoic acid decreased significantly. The reverse experiment showed the opposite. With the development of omics technology, researchers now more often combine multiple parameters to analyze the state of the disease [59]. In 2017, Finnish scientists discovered through metabolomics that high concentrations of indolepropionic acid in serum were potential biomarkers for the development of T2DM, which could mediate its protective effect by maintaining β-cell function [60]. Docosahexaenoic acid (DHA) is an n-3 series of polyunsaturated fatty acids. Current research has shown that DHA has obvious blood glucose lowering and anti-inflammatory effects [61], short-term supplementation of fish oil rich in DHA could significantly reduce Mononuclear cells / macrophage activating factor soluble CD163, triglyceride levels, etc. in patients with T2DM and help to interfere with T2DM and obesity-related complications [62]. Short-chain fatty acids are the main metabolites of dietary fiber fermented by the flora. Among them, acetic acid can be produced from pyruvate in two different ways, one is through intestinal bacteria Acetyl-CoA, and the other is the Wood-Ljungdahl pathway, which can promote insulin, GLP-1, GIP and PYY secretion, promote β-cell growth and regulate inflammation [63]. Studies have found that acetate can prevent obesity and insulin resistance in mice caused by high fat diets. Acetate could reduce the weight gain of mice by 40%, and fasting insulin and leptin levels were significantly reduced [64]. Another study used internal transcription spacer (ITS)-based sequencing to characterize the microbiota of obese and non-obese subjects. The results found a preliminary relationship between obesity and metabolites such as hexanoic acid [65]. 3-Hydroxybutyric acid (3HB) is a ketone body and acts as an indicator of energy balance and a central regulator of energy homeostasis [66]. Studies have shown that the peroxisome proliferator-activated receptor alpha (PPARα) -dependent activation and promotion of fatty acid utilization in the liver induces the production of 3-HB [67], but the relevant mechanism deeply related to obesity-T2DM is not clear yet. It should be further verified and discussed in the later experimental design. In short, in disease-susceptible individuals, the intestinal microbiota became a catalyst for the development of disease. Although the intestinal microbial genome differs among individuals [68], it can modulate multiple functions that affect host metabolism [40, 69], including normal homeostasis [70]. In the previous study, we monitored the intestinal microbiota of diabetic rats for 8 weeks in real time, proving that the role of intestinal microbiota in the development of diabetes provides support [71].

Interestingly, transplanting the intestinal microbiota of ZDF rats with T2DM to heathy LZ rats was not induced them to develop obesity or T2DM, and the structure of the microbiota was not significantly different from that of the control group. In addition, transplantation of the microbiota of LZ rats into ZDF rats only improved the course of the disease to a certain extent, rather than restoring it to normal. This indicates that the microbiota is not the most critical factor leading to disease. Healthy hosts are more capable of regulating homeostasis, and they can correct themselves and return to health, even when the composition of the intestinal community is altered. Regulation of the intestinal microbial composition and function by FMT may only partially affect the intrinsic and complex pathophysiology of obesity and T2DM [72]. The composition and function of the intestinal microbiome are influenced by many factors, and thus, a single FMT is unlikely to cure obesity or T2DM. However, the combination of FMT and personalized probiotics or the addition of “missing” intestinal bacterial strains (drug microbiology) may enhance the effectiveness of conventional treatment strategies [73].

Overall, we demonstrated a role for intestinal microbiota in directing the progression of obesity to T2DM. The intestinal microbiota was more involved in catalyzing progression than in causing disease de novo. At present, our research is still focused on rodents, and whether similar effects occur in humans should be explored. The key role of the intestinal microbiota balance in health has been repeatedly emphasized. We expect that adjusting the dietary structure or providing therapeutic FMT can reduce IR, control obesity, delay and reverse the development of T2DM in the future.

## MATERIALS AND METHODS

### Animals and ethics

In this study, rats were used in animal experiments and approved by the Animal Ethics Committee of Nanjing University of Traditional Chinese Medicine (Grant No. 201103A026). Male 5-year-old ZDF rats (*fa/fa*) and their lean control LZ rats (*fa/+*) were purchased from Vital River Laboratories (China), 20 rats in the donor group and 60 rats in the recipient group, which were raised in the specific pathogen-free animal experiment center of Nanjing University of Chinese Medicine, constant temperature (24±2°C), constant humidity (65%±5%) and accepted a 12h light/dark cycle (7:00 AM-7:00 PM). The animals were fed a radiation sterilized control feed (MD17121, Mediscience, China) or Formulab feed (Purina #5008, Lab diet, USA). Free use of food and autoclaved water. Body weight, abdominal circumference, and random blood glucose were measured weekly during the experiment.

### Preparation of donor group’s microbiota

Rats in the donor group were raised to 9 weeks of age in an optimal environment, and the success of induction of T2DM was evaluated, and they were euthanized after significant difference from the control group. The cecal and colon contents were collected and combined in a sterile test tube, 2 g was stored in a sterile cryotube for the detection of the microbiota. The remaining samples were combined and diluted 20-fold in sterile PBS and centrifuged at 188 ×*g* for 5 minutes [74]. The supernatant was filtered through 70 mm filters and aliquoted for use.

### Antibiotic administration and microbiota transplantation

LZ and ZDF Rats in the recipient group were continuously intragastrically administrated 1 mL broad-spectrum antibiotic mixture containing ampicillin (Cas7177-48-2), gentamicin (Cas1405-41-0), metronidazole (Cas443-48-1), and neomycin (Cas1405-10-3) (1:1:1:1, Solarbio, China) for 10 days [75]. After antibiotic treatment, 16S rRNA was measured in the feces to ensure that the effects of antibiotics on the microbiota were similar. Cecal / colon supernatant (750 μL) from ZDF and LZ donor rats were intragastrically administered to ZDF and LZ recipient rats for 28 consecutive days, and the feces of each group after transplantation were collected. In addition to the above-mentioned rats, other ZDF and LZ rats were intragastrically administered PBS instead of the antibiotic mixture and the donor supernatant. Oral Glucose Tolerance Test (OGTT) and Insulin Tolerance Test (ITT) experiments were performed, and after 12 h of fasting in the evening, the rats were anesthetized with isoflurane, and abdominal aorta blood was taken. Precipitated blood cells (10 μL) were immediately measured for glycosylated hemoglobin (Bio-Rad D-10 glycosylated hemoglobin meter, Bio-Rad, USA). Blood cells were also centrifuged at 1300 ×*g* for 10 min, serum was extracted, and blood lipid levels were measured with a fully automated biochemical analyzer (Chemray 240, Rayto, China). Fasting serum insulin levels (FSI, 10-1250-01, Mercodia, Sweden) and leptin levels (Leptin, Catalogue #PMOB00, R&D Systems, USA) were measured with an enzyme-linked immunosorbent assay. The Homeostasis Model Assessment-Insulin Resistance (HOMA-IR) index was calculated as Fasting Blood Glucose (mmol / L) × FSI (mIU / L) / 22.5. After the rats were sacrificed, the liver was quickly collected, and the contents of the colon were placed in a sterile cryotube and quickly frozen in liquid nitrogen for subsequent analysis.

### 16S rRNA amplification and sequencing and biosignal analysis

As previously described [71], bacterial DNA was extracted from feces and intestinal contents, purified, quantified, and sequenced using the Illumina MiSeq platform. Sequencing libraries were prepared using the TruSeq Nano DNA LT Library Prep Kit (Illumina). The aforementioned sequences were merged by 97% sequence similarity and partitioned by Operational Taxonomic Units (OTU), with QIIME software and UCLUST, a sequence alignment tool. The obtained abundance matrix was used to calculate α-diversity. According to the results of OTU classification and taxonomic status identification, the specific composition of each sample at each taxonomic level can be obtained. Using Mothur, QIIME, and R-software, which we refer to as a Metastats (http://metastats.cbcb.umd.edu/) statistical algorithm, non-weighted UniFrac principal coordinates analysis (PCoA) was used to construct a partial least squares discriminant analysis (PLS-DA) discriminant model to quantify the differences and similarities between samples. The 16S rRNA gene sequence was predicted in KEGG Pathway Database (KEGG), Cluster of Orthologous Groups of Proteins (COG), and RNA families (Rfam), which are three functional spectrum databases, using Phylogenetic Investigation of Communities by Reconstruction of Unobserved States (PICRUSt). Annotation information corresponding to each functional spectrum database was obtained for each sample, and the abundance matrix of predicted functional groups was obtained. A Venn diagram was created. The shared/unique OTU between samples and groups was visualized with a “Venn Diagram” that was created with R software.

### Targeted determination and analysis of metabolites

The samples were homogenized and centrifuged, and the supernatants were combined and subjected to automated sample derivatization and separation using a robotic multi-purpose sample MPS2 (Gerstel, Muehlheim, Germany) with a double head. Microbial metabolites were quantified using gas chromatography using a time-of-flight mass spectrometry (GC-TOFMS) system operating in electron ionization mode (Pegasus HT, Leco Corp., St. Joseph, MO, USA). The reserved solutions of all 132 representative reference chemicals of microbial metabolites were prepared in methanol, ultrapure water or sodium hydroxide solution at a concentration of 5 mg/mL or 1 mg/mL. Internal standards were added to monitor data quality and compensate for matrix effects. The original data generated by GC-TOFMS was processed with proprietary software XploreMET (v2.0, Metabo-Profile, Shanghai, China) [76], to automatically remove baseline values, to smooth and pick peak values, and to align peak signals. XploreMET can perform data processing, interpretation, and visualization. Statistical algorithms were adapted from the widely used statistical analysis software package (R) (http://cran.r-project.org/) using multivariate statistical analysis, such as PLS-DA, OPLS-DA and univariate statistical analysis, including Student’s t-test, the Mann-Whitney-Wilcoxon U-test, Analysis of Variance (ANOVA), and correlation analysis, for data analysis, data and project objectives constituted the best statistical method.

### Scanning and transmission electron microscopy

Colon samples were fixed with 2.5% glutaraldehyde and dehydrated in ethanol twice for 10-15 min each. Samples were immersed in a 1:1 mixture of acetic acid (isoamyl ester): ethanol for 10 min followed by isoamyl acetate for 10 min with shaking. Samples were transferred into a sample basket and placed in the sample chamber of a pre-cooled critical point dryer (K850 critical point dryer, Quorum, UK) in which liquid carbon dioxide was injected to submerge the sample. The sample was pasted with conductive adhesive after gasification with carbon dioxide at elevated temperature. An Ion Sputtering Instrument (108Auto Ion Sputtering Instrument, Cresstington, UK) was used to prepare samples from post-coating endoscopy (SU8010 scanning electron microscope, Hitachi, Japan). For transmission electron microscopy, other samples were dehydrated in a graded series of ethanol (50% ethanol-70% ethanol-90% ethanol-90% ethanol + 90% acetone-90% acetone-100% acetone), embedded, cured, and cut into semi-thin sections (1 μm) and thin sections (70 nm) with an ultramicrotome (EM UC6, Leica, Germany). Photomicrographs were taken after double-staining with 3% uranium acetate-lead citrate (JEM1230 transmission electron microscope, JEOL, Japan).

### Western blotting

Liver tissues were homogenized in RIPA buffer (P0012B, Beyotime, China) supplemented with a mixture of protease inhibitor cocktail (100×) (5871s, CST, USA) and phosphatase inhibitor cocktail (100×) (5870s, CST). The lysates were subjected to Sodium Dodecyl Sulfate-Polyacrylamide Gel Electrophoresis (SDS-PAGE) and blotted with the following antibodies: phospho-Janus Kinase Signal Transducers 2 (JAK2) (Tyr1007/1008) (3776S, CST, 1:1000), JAK2 (3230S, CST, 1:1000), phospho-Insulin Receptor Substrate 2 (IRS2) (Ser371) (Ab3690, Abcam, 1:1000), IRS2 (4502S, CST, 1:1000), phospho-Protein Kinase B (Akt) (Ser473) (4060s, CST, 1:1000), Akt (9272s, CST, 1:1000), Forkhead Transcription Factor 1 (FOXO1) (2880S), CST, 1:1000), and β-actin (3700S, CST, 1:1000). The membranes were incubated with secondary antibodies conjugated to HRP (BA-1054/BA1050, Boster, China, 1:2000). The immunoreactive bands were treated with a chemiluminescence solution (ECL, Tanon, China) and detected with X-ray films. The blots were visualized with an Amersham Imager 600 (General Electric Company, USA) and analyzed with ImageQuant TL 1D software (GE Healthcare, USA).

### Data and statistics

The data for the physiological characteristics of the rats were expressed as the mean ± standard deviation (SD). Statistical analysis of differences between the different groups was performed with two-way ANOVA and then tested using Tukey’s true significant differences test. When only two groups were compared, Students’ t-test was used. The correlation between data for the physiological characteristics and different microbiota or metabolites was tested using Spearman correlation analysis. All analyses were performed using Prism 8.0 (GraphPad, La Jolla, CA, USA) software.

### Data availability

The datasets generated in this study are available through the NCBI Sequence Read Archive (accession number SRP227423).

## Supplementary Material

Supplementary Figures

Supplementary Table 1
