# Supplementary material for: Fecal microbiota transplantation alters the susceptibility of obese rats to type 2 diabetes mellitus
Source: Aging (Albany NY). 2020 Sep 12;12(17):17480–502. doi: 10.18632/aging.103756 (PMC7521520; doi:10.18632/aging.103756)
Supplement: Supplementary Figures [file aging-12-103756-s002..pdf]

SUPPLEMENTARY FIGURES

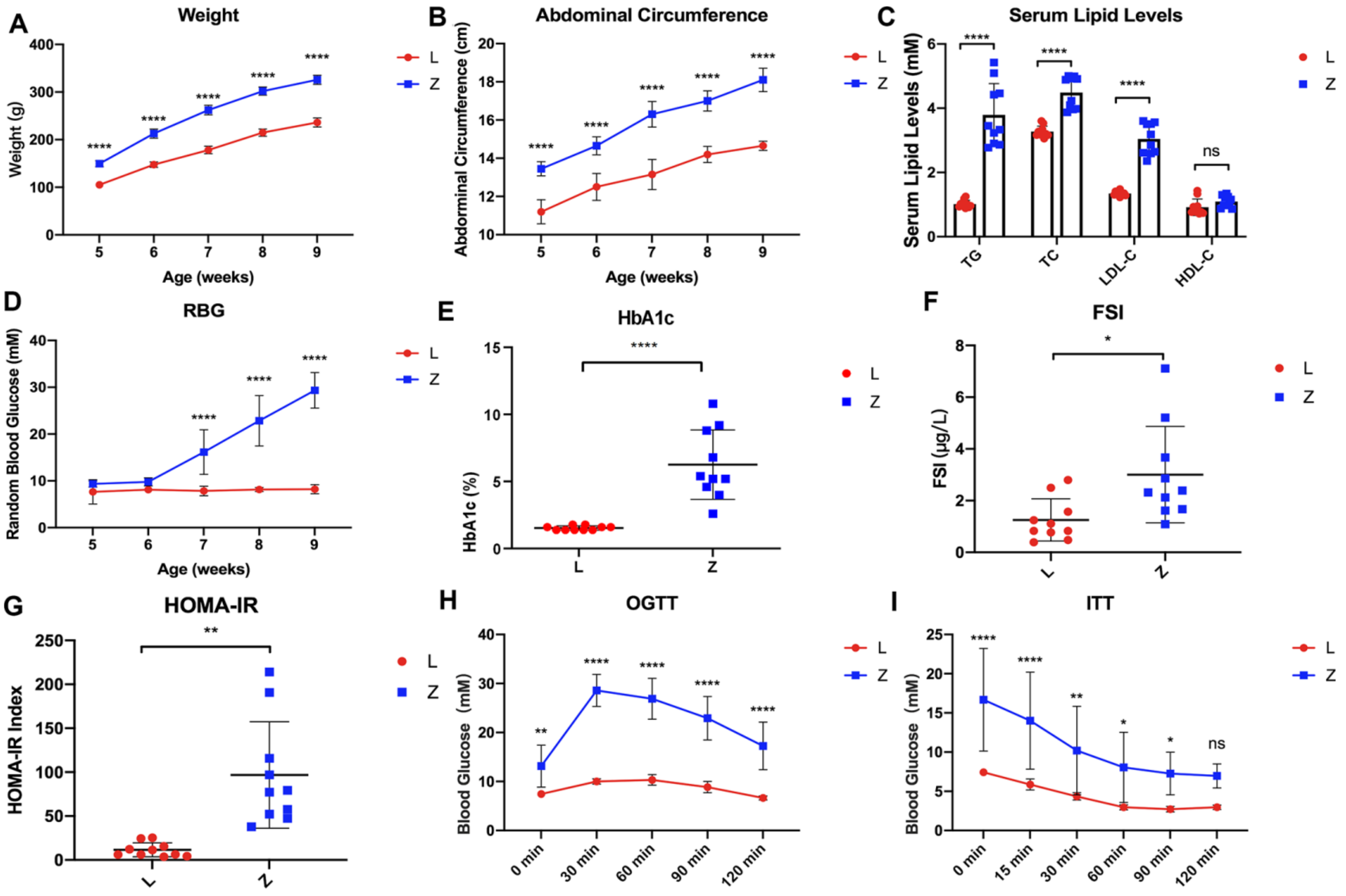

Supplementary Figure 1. Basic information of the donor group rats. (**A**) Body weight (g; Time: $F_{4, 45}$ =1254, $P < 0.0001$; Group: $F_{1, 45}$ = 1972, $P < 0.0001$; Interaction: $F_{4, 45}$ = 26.78, $P < 0.0001$); (**B**) Abdominal circumference (cm; Time: $F_{4, 45}$ =179.3, $P < 0.0001$; Group: $F_{1, 45}$ = 535.6, $P < 0.0001$; Interaction: $F_{4, 45}$ = 4.437, $P = 0.0042$); (**C**) TG (mM; t = 13.79, $P < 0.0001$), TC (mM; t = 6.024, $P < 0.0001$), LDL (mM; t = 8.414, $P < 0.0001$), and HDL (mM; t = 0.8776, $P > 0.05$); (**D**) Random blood glucose (mM; Time: $F_{4, 45}$ = 48.78, $P < 0.0001$; Group: $F_{1, 45}$ = 304.2, $P < 0.0001$; Interaction: $F_{4, 45}$ = 48.17, $P < 0.0001$); (**E**) Glycosylated hemoglobin (%; t = 5.757, $P < 0.0001$); (**F**) Fasting serum insulin (μg / L; t = 2.717, $P < 0.05$); (**G**) HOMA-IR index (t = 4.437, $P < 0.01$); (**H**) Oral glucose tolerance (mM; Time: $F_{4, 45}$ = 37.46, $P < 0.0001$; Group: $F_{1, 45}$ = 426.5, $P < 0.0001$; Interaction: $F_{4, 45}$ = 12.86, $P < 0.0001$); (**I**) Insulin tolerance (mM; Time: $F_{5, 54}$ = 14.72, $P < 0.0001$; Group: $F_{1, 54}$ = 92.95, $P < 0.0001$; Interaction: $F_{5, 54}$ = 1.799, $P = 0.1287$) comparison of donor LZ and ZDF rats. n = 10. Statistical analysis was performed with two-way ANOVA in (**A**, **B**, **D**, **H**, **I**) and Student's t-test followed by Tukey's test in (**C**, **E**, **F**, **G**). *$P < 0.05$, **$P < 0.01$, ***$P < 0.001$, ****$P < 0.0001$. The data are expressed as the mean ± SD.

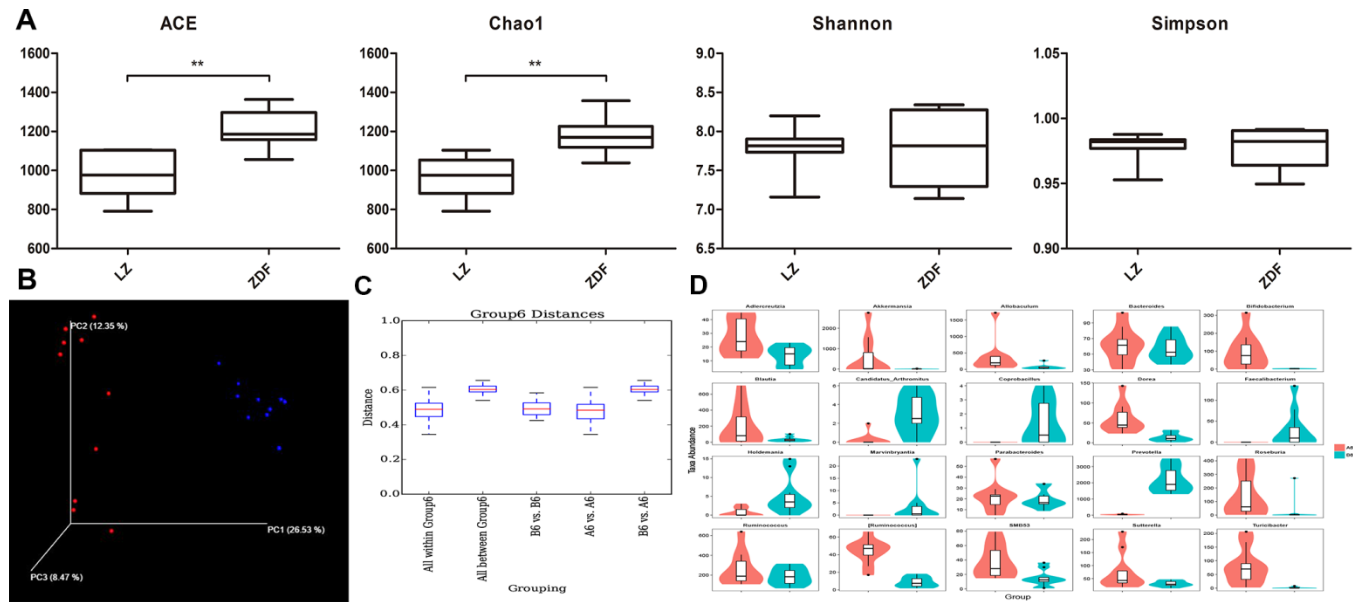

Supplementary Figure 2. Intestinal microbiota structure of the donor group. (**A**) Comparison of α-diversity between donor LZ and ZDF rats (ACE: t = -4.121, *P* = 0.001; Chao1: t = -3.963, *P* = 0.002; Shannon: t = -1.600, *P* = 0.136; Simpson: t = 0.292, *P* = 0.774); (**B**) Three-dimensional sequence diagram of samples of Unweighted UniFrac PCoA analysis of LZ and ZDF rats (n = 10); (**C**) Box plot of the difference in UniFrac distance values for different groups corresponding to the two groups of rats (n = 10); (**D**) Violin map of the abundance distribution of the top 20 taxa with the most significant difference between the sample groups (n = 10). Red, LZ group; Blue, ZDF group. Statistical analysis was performed with Student's t-test, and Monte Carlo permutation test, or Student's t-test followed by Tukey's test. *$P < 0.05$, **$P < 0.01$, ***$P < 0.001$, ****$P < 0.0001$. The data are expressed as the mean ± SD.

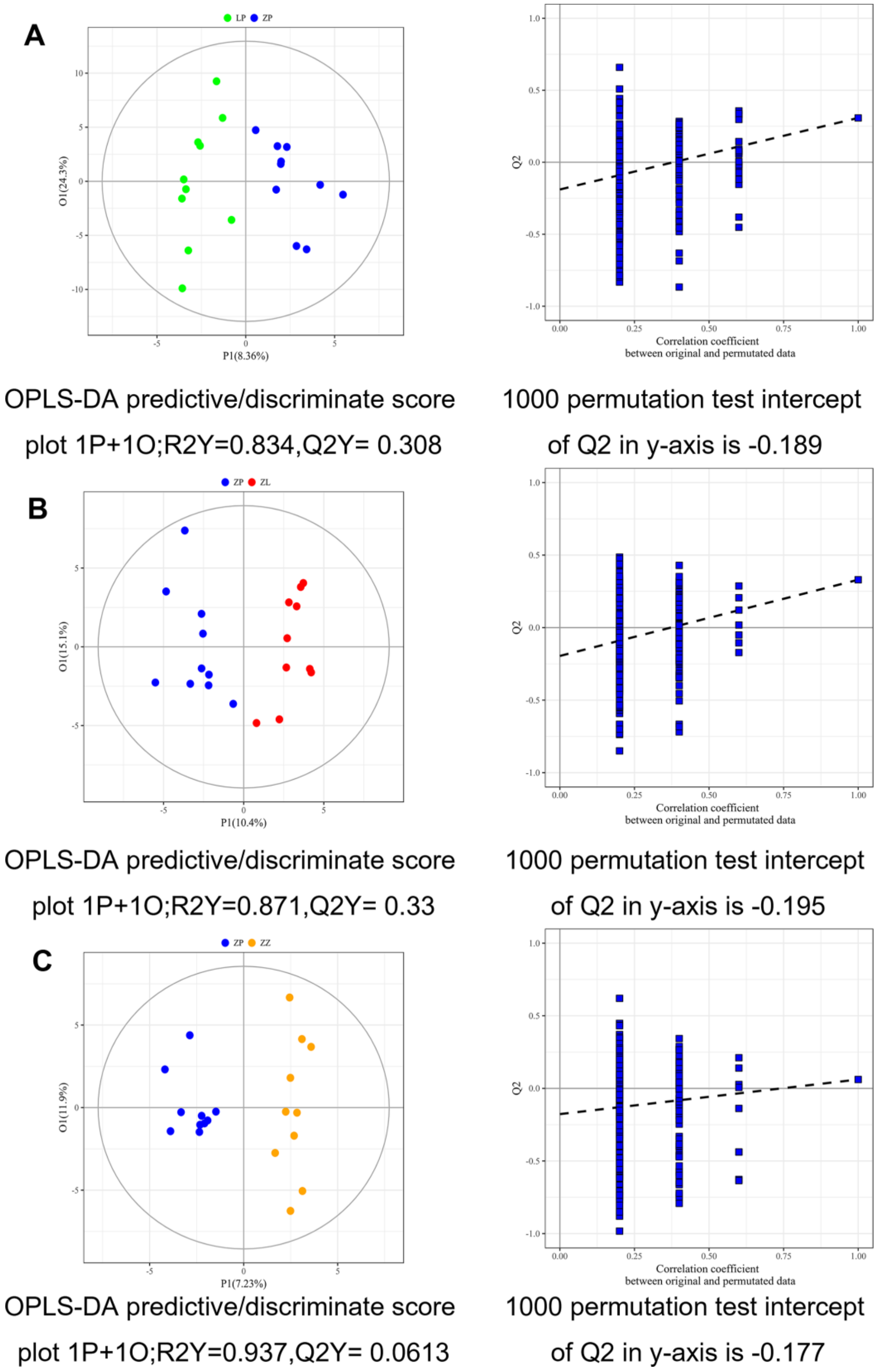

Supplementary Figure 3. Orthogonal partial least squares discriminant analysis (OPLS-DA) among the normal group, model group, and intervention group. (**A**) Diagnostic parameters and regression curves of pairwise comparison between the L-P group and Z-P group; (**B**) the Z-P group and Z-Lg group; (**C**) the Z-P group and Z-Zg group.
