# Supplementary material for: Fecal microbiota transplantation alters the susceptibility of obese rats to type 2 diabetes mellitus
Source: Aging (Albany NY). 2020 Sep 12;12(17):17480–502. doi: 10.18632/aging.103756 (PMC7521520; doi:10.18632/aging.103756)
Supplement: Supplementary Table 1 [file aging-12-103756-s001..pdf]

SUPPLEMENTARY TABLE

Supplementary Table 1. Different genera corresponding to the intestinal microbiota of the donor and recipient.

Group	Dominant group	Taxa	*P*-value	*q*-value
Donor	LZ	*Adlercreutzia*	0.000999	0.000404
	LZ	*Allobaculum*	0.000999	0.000404
	LZ	*Bacteroides*	0.000999	0.000404
	LZ	*Dorea*	0.000999	0.000404
	LZ	[*Ruminococcus*]	0.000999	0.000404
	LZ	*Turicibacter*	0.000999	0.000404
	LZ	*Bifidobacterium*	0.001998	0.000588
	LZ	*SMB53*	0.001998	0.000588
	LZ	*Sutterella*	0.001998	0.000588
	LZ	*Blautia*	0.016983	0.003667
	LZ	*Parabacteroides*	0.018981	0.003775
	LZ	*Roseburia*	0.020979	0.003775
	LZ	*Ruminococcus*	0.020979	0.003775
	LZ	*Akkermansia*	0.025974	0.004206
	ZDF	*Coprobacillus*	0.000999	0.000404
	ZDF	*Prevotella*	0.000999	0.000404
	ZDF	*Faecalibacterium*	0.006993	0.001887
	ZDF	*Candidatus_Arthromitus*	0.010989	0.002738
	ZDF	*Marvinbryantia*	0.015984	0.003667
	ZDF	*Holdemania*	0.023976	0.004087
Recipient	LZ	*Lactobacillus*	0.000999	0.009365
	LZ	*Roseburia*	0.012827	0.101737
	LZ	*Coprococcus*	0.015984	0.037874
	LZ	*Allobaculum*	0.000999	0.037204
	LZ	*Rothia*	0.004995	0.032776
	LZ	*Clostridium*	0.003996	0.043003
	ZDF	*Bacteroides*	0.002997	0.074407

non-parametric t-test for testing.
